# Spatiotemporal Variations in Gastric Cancer Mortality and Their Relations to Influencing Factors in S County, China

**DOI:** 10.3390/ijerph16050784

**Published:** 2019-03-04

**Authors:** Cheng Cui, Baohua Wang, Hongyan Ren, Zhen Wang

**Affiliations:** 1 State Key Laboratory of Resources and Environmental Information System, Institute of Geographic Sciences and Natural Resources Research, Chinese Academy of Sciences, Beijing 100101, China; cuic@lreis.ac.cn (C.C.); wangz.16b@igsnrr.ac.cn (Z.W.); 2 College of Resources and Environment, University of Chinese Academy of Sciences, Beijing 100049, China; 3 National Center for Chronic and Non-communicable Disease Control and Prevention, Chinese Center for Disease Control and Prevention, Beijing 102206, China

**Keywords:** gastric cancer mortality, GeoDetector, spatiotemporal variation, environmental improvement, socioeconomic development

## Abstract

Increasingly stricter and wider official efforts have been made by multilevel Chinese governments for seeking the improvements of the environment and public health status. However, the contributions of these efforts to environmental changes and spatiotemporal variations in some environmental diseases have been seldom explored and evaluated. Gastric cancer mortality (GCM) data in two periods (I: 2004–2006 and II: 2012–2015) was collected for the analysis of its spatiotemporal variations on the grid scale across S County in Central China. Some environmental and socioeconomic factors, including river, farmlands, topographic condition, population density, and gross domestic products (GDP) were obtained for the exploration of their changes and their relationships with GCM’s spatiotemporal variations through a powerful tool (GeoDetector, GD). During 2004–2015, S County achieved environmental improvement and socioeconomic development, as well as a clear decline of the age-standardized mortality rate of gastric cancer from 35.66/10^5^ to 23.44/10^5^. Moreover, the GCM spatial patterns changed on the grid scale, which was spatially associated with the selected influencing factors. Due to the improvement of rivers’ water quality, the distance from rivers posed relatively larger but reversed impacts on the gridded GCM. In addition, higher population density and higher economic level (GDP) acted as important protective factors, whereas the percentage of farmlands tended to have adverse effects on the gridded GCM in period II. It can be concluded that the decline of GCM in S County was spatiotemporally associated with increasingly strengthened environmental managements and socioeconomic developments over the past decade. Additionally, we suggest that more attentions should be paid to the potential pollution caused by excessive pesticides and fertilizers on the farmlands in S County. This study provided a useful clue for local authorities adopting more targeted measures to improve environment and public health in the regions similar to S County.

## 1. Introduction

Rapid and huge economic growth has occurred in China during the four decades of reform and opening up. However, China is also facing a huge burden of disease during the process of economic and social development. More than 2.8 million people died of cancer in 2015, and gastric cancer is the second leading cause of cancer death in China, causing the death of nearly 500,000 people in 2015 [1], and it has been a serious threat to sustainable development due to its huge health and finical burden on the country and individuals in China [2].

As a complex chronic non-communicable disease, gastric cancer is closely associated with many risk factors on the global, national, provincial, municipal, and county scale [3,4,5,6,7,8,9,10,11]. Personal lifestyles, such as high intake of salt, excessive alcohol consumption, and insufficient fruits and vegetables intake were strongly associated with gastric cancer [3,4,5,6]. Long-term exposure to water pollution caused by wastewater, sewage, and excessive application of fertilizers and pesticides from industrial and agricultural production also posed important impacts on the epidemiological status of gastric cancer [7,8,9]. In addition, positive family history of this disease and *Helicobacter pylori* (*H. pylori*) infection both increased the risk of developing gastric cancer [10,11].

The influences of these risk factors on gastric cancer were deeply explored through various methods, such as meta-analysis [3], spatial correlation analysis [7,8], Delphi approach [11], and so on. Among these methods, GeoDetector (GD) is a powerful tool to analyze the effect of several independent variables on the spatial distribution of the dependent variable based on spatial variance analysis [12], since spatial heterogeneity is a major issue in geographical phenomena [13]. It was initially applied to analyze the effects of driving determinants on local disease such as neural tube defects [14]. Due to its good applicability, GD not only has an extensive application on public health issues [15,16,17], but is also an exploration in the fields of land use, ecology, regional economy, and so on [12].

In recent years, public awareness about the increasingly serious environmental pollution has been greatly raised due to its important impacts on public health [18,19]. Although China has completed rapid development and huge socioeconomic achievements, the sustainable development mode, in particular the improvement of public health status, has been set as a basic requirement and national strategy [20]. As a result, research has been gradually focusing in on the evaluation of the influences of environmental factors, as well as their changes, on the incidences, and/or mortality of some chronic non-communicable diseases, since the environmental protection measures and hygienic interventions have been strengthened [21,22,23].

In the Central China, S County is well-known for its environmental deterioration and a distinct increase of gastric cancer mortality (GCM) over the past decades [24]. Fortunately, the continuous development of the social economy, increasingly strict environmental managements, and strengthened comprehensive cancer interventions had been conducted in this county by the central, provincial, and local government since 2007 [25,26,27]. However, the evaluation on the contribution of these measures to the status of GCM was not conducted in time.

Therefore, our study aimed to (1) investigate the spatial and temporal patterns of GCM in S County during 2004–2015, and to (2) explore the associations between GCM and environmental conditions and socioeconomic development, as well as their changes through the GD method. This study would provide some useful clues for local authorities realizing their efforts made for enhancing environment and improving public health.

## 2. Materials and Methods

### 2.1. Study Area

**Figure 1:**
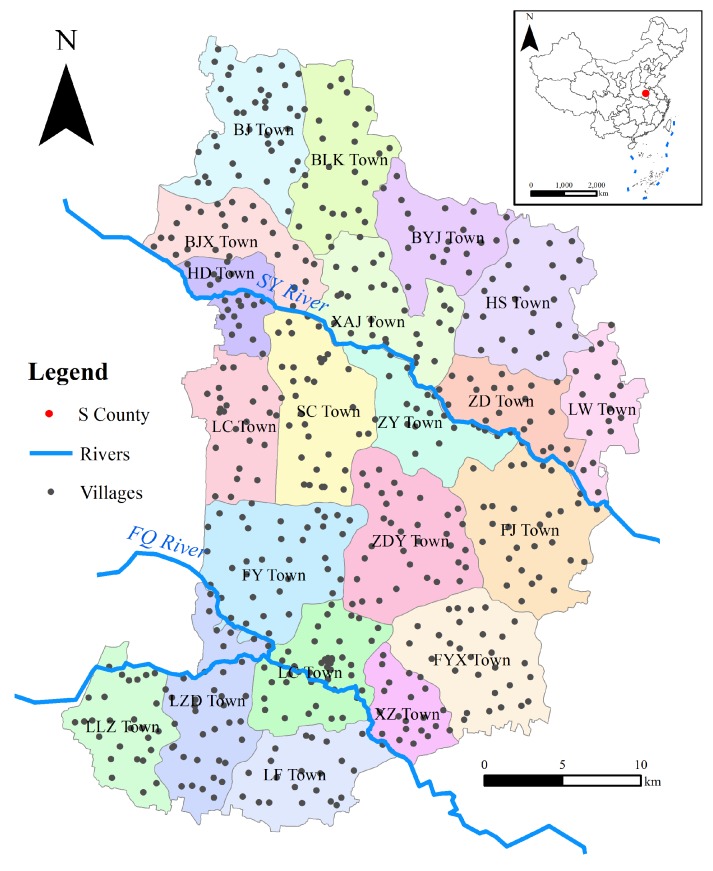
Illustration of S County.

S County (‘S’ derives from the initial of the county name, we have to conceal the full name of some geographical vocabularies related to S County for some reasons), located in Central China, is a ‘famous’ region with both serious environmental pollution and high GCM [7,28]. S County includes 21 towns with a total area of 1080 km^2^ (Figure 1). S County is generally flat (altitude 35–50 m), and it is gently inclined and slightly descends from northwest to southeast. The region is dominated by warm temperatures and a continental monsoon climate with an annual average temperature of 14.5 °C and an annual rainfall of about 700 mm/m². There are two rivers, the SY and FQ rivers, flowing through this county from northwest to southeast.

### 2.2. Data Collection

The death cases of gastric cancer (10th revision of the International Classification of Diseases code: C16) in 2004–2006 (Period I) and 2012–2015 (Period II) were derived from retrospective cause of death survey data and death surveillance data conducted by Chinese Center for Disease Control and Prevention. On the basis of village population in 2004 and the registered population growth of this county during 2004 to 2012 [29], the village population in 2012 was obtained. The age-standardized mortality rate was adjusted by Chinese standard population in 2000. To avoid the instability of GCM caused by irregular and non-uniformity of village administrative units [30,31], we used a 2 × 2 km^2^ grid as a basic statistical unit, which has been proven to be an appropriate analysis scale for S County [24]. In total, there were 260 grids across this county. For all geographical units, GCM data ware spatially smoothed using an empirical Bayesian smoothing method in GeoDa (GeoDa 1.12.1, April 2018, the Center for Spatial Data Science (The University of Chicago), Chicago, IL, USA) before conducting spatial and statistical analysis [32].

**Figure 2:**
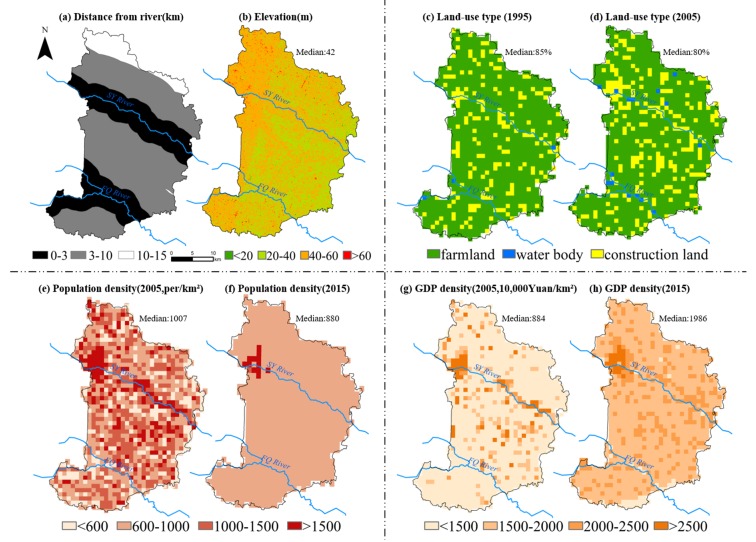
Five environment proxy variables of S County, including (**a**) distance from the river; (**b**) elevation (30 × 30 m^2^); (**c**)–(**d**) land-use type (1 × 1 km^2^); (**e**)–(**f**) resident population density (1 × 1 km^2^); (**g**)–(**h**) GDP density (1 × 1 km^2^). ’Median’ in (**b**)–(**h**) is the corresponding median value of proxy variables on grid scale.

As can be seen in Table 1 and Figure 2, the data of some proxy variables were collected for indicating these influencing factors during these two periods in S County, including human-made pollution, physical environment, and socioeconomic level [14,15]. Given that surface water pollution is an important environmental risk factor [7], the Euclidean distance between the grid center from the nearer river (SY or FQ river) was calculated for indicating the contact opportunity between residents and rivers. As S County is featured by agricultural production, the percentage of farmlands was derived from the land use data for indicating the potential pollution caused by excessive application of pesticides and fertilizers in this area [33]. As for the physical environment, topography plays an important role in the migration of carcinogens [34], and GCM may vary significantly at different altitudes [24]. Meanwhile, resident population density and gross domestic product (GDP) were used to measure the local socioeconomic level, which may affect family income, household sanitation, eating habits, and medical service level [35,36]. The gridded resident population density and GDP data were disaggregated from the county level based on multivariate statistical models [37,38].

### 2.3. Spatial Analysis Methods

#### 2.3.1. Spatial Autocorrelation Analysis

Spatial autocorrelation analysis is commonly used to explore the spatial patterns of disease incidence or mortality. In this study, we used Moran’s Indices to measure the degree of spatial autocorrelation [40], which is calculated as follows:

(1) $$I=\frac{n\sum_{i=i}^{n}\sum_{j=1}^{n}\omega_{ij}\left(x_{i}-\bar{x}\right)\left(x_{j}-\bar{x}\right)}{\sum_{i=i}^{n}\sum_{j=1}^{n}\omega_{ij}\sum_{i=1}^{n}{\left(x_{i}-\bar{x}\right)}^{2}}$$

where n is the number of grids; $x_{i}$ and $x_{j}$ stand for the GCM of grid i and grid j; $\bar{x}$ is the average value of GCM; and $\omega_{ij}$ is the spatial weight between grid i and grid j that can be defined by the contiguity of grids. Moran’s I value falls between −1 and 1. A high positive Moran’s I indicated a spatial clustering, that is, adjacent grids had similar levels of GCM, whereas a low negative value implied a tendency toward dispersal. When Moran’s I is around zero, the value indicated spatial randomness.

#### 2.3.2. Hot Spot Analysis

On the basis of the existence of spatial autocorrelation, hot spot analysis further captured the detailed spatial patterns by means of locating hot or cold clusters of GCM. We selected Getis-Ord Gi* to identify the locations of statistically significant hot and cold spots according to formula (2):

(2) $$G_{i}^{*}=\frac{\sum_{j=1}^{n}\omega_{ij}x_{j}-\bar{X}\sum_{j=1}^{n}\omega_{ij}}{S\sqrt{\frac{\left[n\sum_{j=1}^{n}\omega_{ij}^{2}-{\left(\sum_{j=1}^{n}\omega_{ij}\right)}^{2}\right]}{n-1}}}$$

where $x_{j}$ is the GCM value for grid j; $\omega_{ij}$ stands for the spatial weight between grid i and grid j; n means the number of grids; and:

(3) $$\bar{X}=\frac{\sum_{j=1}^{n}x_{j}}{n}$$

(4) $$S=\sqrt{\frac{\sum_{j=1}^{n}x_{j}^{2}}{n}-{\left(\bar{X}\right)}^{2}}$$

The Gi* statistic is a Z-score, and therefore, no further calculation is needed. For statistically significant positive Z-score, the larger the Z-score is, the more intense the clustering of high GCM (i.e., a hot spot). For statistically significant negative Z-score, the smaller the Z-score is, the more intense the clustering of low GCM (i.e., a cold spot). By conducting a hot spot analysis on GCM during the two specified periods, we were able to visually understand the spatiotemporal changes in GCM. We performed spatial autocorrelation and hot spot analysis on the ArcGIS 10.2 platform (ESRI, Redland, CA, USA).

#### 2.3.3. GeoDetector

In this study, GD was used to explore the determinants of spatial variations of GCM in S County during two periods. The basic idea of the method was to compare the spatial consistency of the continuous response variable versus the categorical explanatory variables, and on this basis, to quantify the interpretation of the explanatory variables in relation to the response variable. Both categorical and discretized continuous explanatory variable can be analyzed by GD, while the classic regression model has limitations when it comes to dealing with categorical data [14]. Another advantage is that GD could be used to study the interaction impact of multi factors.

In this study, the response variable is the gridded GCM, and the explanatory variables are the proxy variables as listed in Table 1. The correlation strength between factor X and attribute Y is measured by q-statistic, which is expressed by the following equation:

(5) $$q=1-\frac{1}{N\sigma^{2}}\overset{L}{\underset{h=1}{\sum }}N_{h}\sigma_{h}^{2}$$

where h = 1, 2 … indicates that attribute Y is stratified by factor X (categorical variable or discretized continuous variable); N and $N_{h}$ mean the number of total grids and grids in stratum h, respectively; $\sigma^{2}$ and $\sigma_{h}^{2}$ stand for the variance of attribute Y in all grids and grids in stratum *h*, respectively. The value of q ranges from 0 to 1. The higher the q value, the stronger the explanatory power of factor X to attribute Y. Moreover, the interaction detector could reveal the interactive influence of multi factors. A detailed introduction and computing tool are available at http://www.geodetector.org/.

## 3. Results

### 3.1. Spatiotemproal Variations of Influencing Factors

According to the surface water quality data derived from the state-control stations set for FQ and SY Rivers (Table S1 in Supplementary Files), the water environment of these two rivers has been obviously improved in the past years. As a typical region featured by agricultural production, S County has about 90500 and 85800 ha farmlands in 1995 and 2005, respectively, accounting for more than 70% of the total lands. From 1995 to 2005, about 5.2% farmlands were transformed into other land-use types (Figure 2c,d), which were mainly distributed in the region along the SY river and between the two rivers. The area of construction land increased by over 20 km^2^ in the region between the two rivers. There was a decline in the number of the resident population because of labor export in last decade [41], due to increasing urbanization of population as more and more rural people concentrated living in towns with better economic conditions for better living conditions. As a result, the distribution of resident population density presented clear spatial changes across this county (Figure 2e,f). Meanwhile, the economic level indexed by GDP possessed an outstanding increase from 10.9 billion (2005) to 21.4 billion (2015). Moreover, it was spatially differentiated across this county, the HD Town and BTX Town were the relatively developed areas across S County (Figure 2g,h). These results showed that S County achieved clear socioeconomic development and environmental improvement during 1995–2015.

### 3.2. Spatiotemporal Variations of GCM

**Figure 3:**
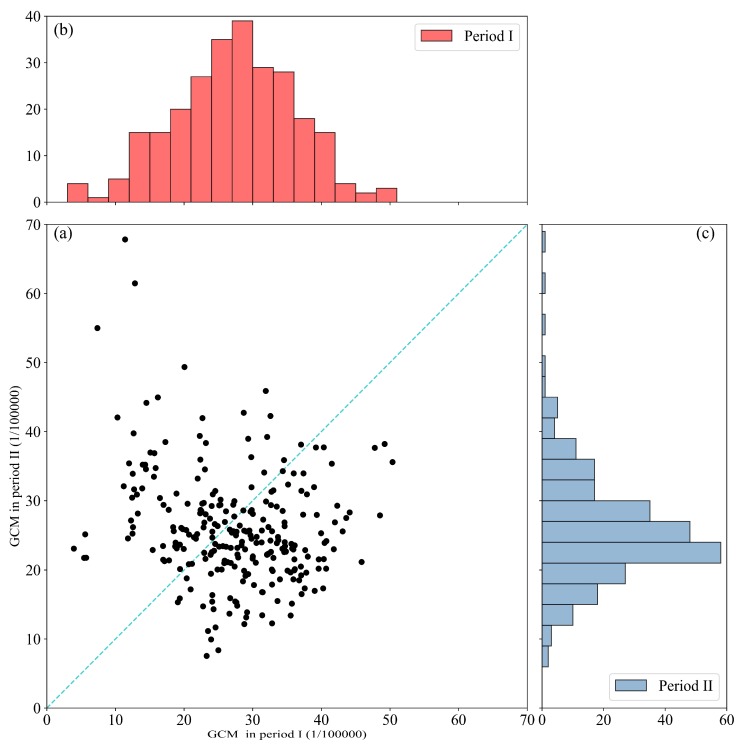
Gastric cancer mortality (GCM) in two periods. (**a**) Scatter plot of GCM; the blue dotted line is the 1:1 line. (**b**) Histogram of GCM in period I, the null hypothesis of normality was retained at the 0.05 level of significance. (**c**) Histogram of GCM in period II, the null hypothesis of normality was rejected at the 0.05 level of significance.

The age-standardized mortality rate of gastric cancer of S County displayed a decrease from 35.66/10^5^ (period I) to 23.34/10^5^ (period II). As illustrated in Figure 3, the gridded GCM was approximately described by a normal distribution in period I, and it turned into non-normal distribution and was right-skewed in period II. There were more than 60% points (the grids) below the 1:1 line (Figure 3), which indicates that the GCM of these grids declined while the others increased in 2004–2015. Meanwhile, the differences of the gridded GCM among these two periods were significant (*p* < 0.1) according to the results of the paired T-test. These results showed that the GCM of S County possessed a decline trend during 2004–2015.

**Figure 4:**
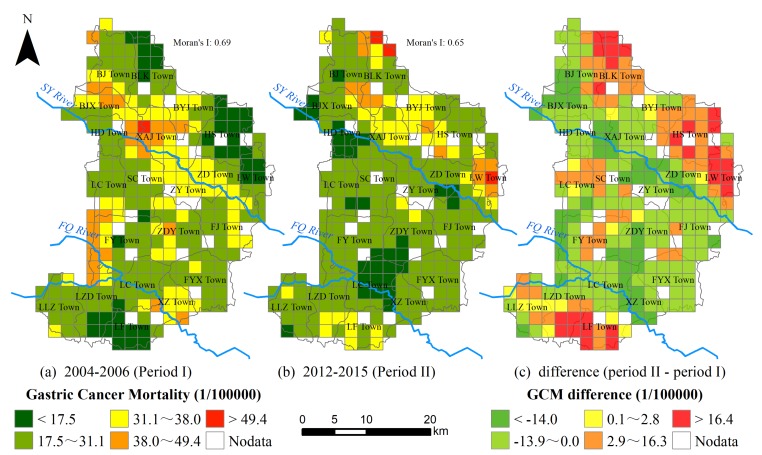
Spatial variation of GCM. (**a**) GCM spatial pattern in period I; (**b**) GCM spatial pattern in period II, the Moran’s I is significant at 0.01 level; (**c**) the GCM difference between period I and period II (the grid without villages points were not taken into account).

In S County, GCM was spatially featured on the grid scale in both periods. According to the Moran’s I as given in Figure 4, the gridded GCM was spatially clustered. Meanwhile, the grids with high GCM in the period I were mainly distributed along with two rivers, especially in the towns of XAJ and ZY around the SY River. In comparison, these grids tended to be away from these rivers in 2012–2015, resulting in a region with relatively lower gridded GCM between FQ and SY River. In addition, the grids with increasing GCM were mostly located in the towns away from the rivers including BLK Town in the northeastern corner, HS Town and LW Town in the east, and LF Town in the south as illustrated in Figure 4c. These results displayed that the spatial patterns of the gridded GCM had changed obviously.

**Figure 5:**
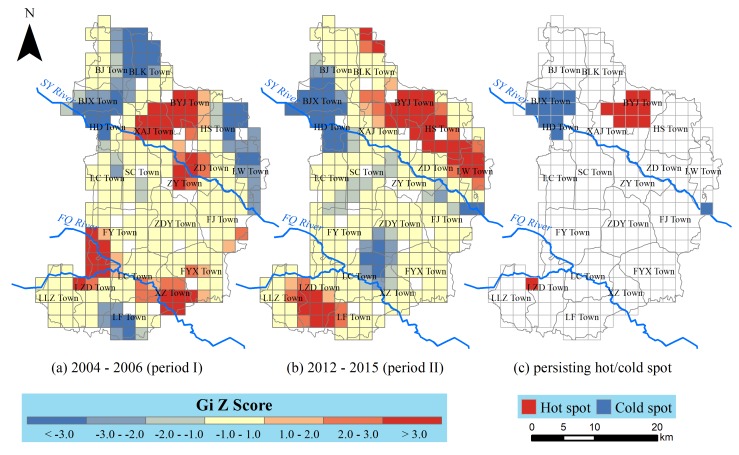
Result of hot spot analysis in terms of Gi Z score. (**a**) Hot/cold spot in Period I; (**b**) hot/cold spot in period II; (**c**) hot/cold spot both in Period I and Period II.

Moreover, the spatiotemporal variations in the gridded GCM were further confirmed through the hotspot analysis. As illustrated in Figure 5, the hotspot grids (Z score above 2.0) in both periods, as well as some persisting hotspot grids, were identified. In period II, the amount of hotspot grids increased from 42 to 46 and tended to be away from FQ and SY River. As a result, the persisting hotspot grids were respectively distributed in BYJ Town (9 grids) and LZD Town (1 grid), which deserved continuous attention in the future.

### 3.3. Relationships between GCM and Influencing Factors

By means of the GD method, the potential determinants were identified for the spatial variations in the gridded GCM across S County (Table 2). Not only the spatial variations of the factors across S County changed between the two periods (Figure 2), but also their effect on gridded GCM changed obviously. In these two periods, the variable indexed by the distance from the river was always an important factor, although its impacts decreased in the II period. In comparison, the elevation failed to impose impacts, and the other three variables indexed by the percentage of farmlands, population density, and GDP significantly affected the spatial variations in the gridded GCM across S County only in the II period. As a result, the powers of these factors could be ranked as following in period II: human-made pollution > socioeconomic level > physical environment. These results displayed that these selected variables had important effects to various degrees on the spatial variations in the gridded GCM across S County.

In addition, the statistically significant difference in the average GCM between different amounts of strata was identified by the GD method (Table 3). In Period I, the closer to the river, the higher the GCM (Table 3). The effects of the distance from river on gastric cancer were reversed in Period II. There was no significant GCM difference at different elevation, which was fairly level with little variations across S County. The other variables showed spatial stratified heterogeneity only in Period II: GCM was high in the areas with a high percentage of farmlands; regions with more intensive population and GDP were accompanied by a lower GCM. Therefore, these variables’ influences on the spatial variations in the gridded GCM had distinctly changed over the past decades.

## 4. Discussion

At present, environmental changes and status of public health are two important public issues in China, especially in some regions (i.e., S County) with historic environmental pollution and serious public health. The spatiotemporal variations in the GCM and their relationships with environmental changes and socioeconomic status across S County were explored on a fine spatial scale in this study. Several interesting findings were achieved and would provide useful clues for local authorities to evaluate the measures of environmental protection and socioeconomic development timely.

There was a remarkable decrease (37.4%) observed for the age-standardized mortality rate of gastric cancer in S County during 2004–2015. We think that the decline was probably related with the environmental improvement and socioeconomic development in this county. To our knowledge, quite a few small factories such as textile mills were shut down in the past decades, and similar policies were conducted in the upstream of SY and FQ River [23,25], which resulted in the environmental improvement, especially the surface water quality of SY and FQ River in S County (Table S1 in Supplementary Files). Meanwhile, the decline of resident population reduced the domestic pollution [42], and increasing urbanization of population and the development of economic guaranteed the implement of relevant interventions such as drinking water safety project [27,43]. In particular, the percentage of local population covered by the centralized water supply (i.e., the level of drinking water safety) in this county had been raised from less than 30% (2005) to more than 85% in 2015 [8,44]. Moreover, economic growth is always accompanied by the improvement of people’s living standards. Improvement of household sanitation, healthy eating habits, and the use of refrigerators all reduced the risk of developing gastric cancer [6,45,46]. In addition, the remarkable decrease was larger than the national level (20.6%) in the corresponding period, although S County still possessed a higher mortality rate (23.34/10^5^) of gastric cancer than the national level (19.62/10^5^) at present [7,47]. In a word, these results were encouraging for local authorities who had made great efforts, and then they could hand in a passable answer sheet for the public.

Similarly, the spatial patterns of the gridded GCM changed obviously along with spatially differentiated socioeconomic development and environmental improvement in S County during 2004–2015. Among current proxy variables, the percentage of farmlands possessed an adverse effect on this disease, which was probably caused by excessive application of pesticides and fertilizers in this agricultural region [48]. Moreover, the distance to river made a relatively large contribution to the spatial variations in the gridded GCM, which was probably due to the historic status of both environment and public health. Before 2005, S County was well-known for its serious surface water pollution and some famous “cancer villages” distributed along with SY and FQ River [24], for which local authorities had implemented some strict environmental management (e.g., centralized water supply projects) in the towns along with these rivers [26]. However, the total contributions of current influencing factors were not large enough to fully interpreting the present spatial variations across S County. One reasonable explanation is that there may be one or more missing crucial variables (i.e., the town/village-level hygienic intervention) in this study. Another is that more information about the income level on the village/family scale was not adequately reflected by the economic level (GDP) employed in current study. In future investigations, more typical potential variables should be obtained for the evaluation. Nevertheless, these selected proxy variables have made clear contributions to the spatial patterns of GCM on the grid scale over the past decade.

A preliminary trial was conducted in our study to characterize the spatiotemporal variations of GCM, and to evaluate their relations to some proxy variables indicating some environmental and socioeconomic changes in S County. To our knowledge, S County suffered from high mortality rates of other digestive tract cancers (e.g., liver, esophagus, etc.), which were closely associated with local environmental conditions over the past decades [7]. Moreover, there were several counties or districts possessing similar public health problems and environmental pollution, especially in Central China [28]. We think that this study exhibited a feasible paradigm for local authorities who would submit their answer sheets on environmental management and public health status.

Several limitations of our study warrant mention. First, many more reliable variables, such as the village/household-level income conditions, and town/village-level hygienic interventions on this disease should be obtained and employed for more detailed analysis of spatiotemporal change of GCM in S County. Second, the contributions of selected variables to the spatial heterogeneities of GCM should be spatially characterized for each grid by using some spatiotemporal models (e.g., geographically weighted regression (GWR), temporal GWR, etc.) in the future, although the GD method has quantified these variables’ effects in the present study.

## 5. Conclusions

The decline of GCM and its changes of spatial patterns in S County benefited from the environmental improvement and socioeconomic development. We suggest that the potential pollution derived from agricultural production should be effectively controlled, and that the local government should further promote the sustainable development mode in terms of coordinated the relationship between environment and the economy to maintain the positive situation. The study provided informative knowledge for other typical regions which were similar to S County.

## Figures and Tables

**Table 1:** Overview of proxy variables and data source.

Environmental Factors	Proxy Variables	Dataset	Time	Resolution	Source
Human-made pollution	Distance from river	—	—	—	—
	Percentage of farmlands	Land use data	1995, 2005 *	1 km	www.resdc.cn
Physical environment	Elevation	Global Digital Elevation Model (V2)	2009	30 m	www.gscloud.cn
Socioeconomic level	Population density	Gridded resident population density	2005, 2015	1 km	www.resdc.cn
	Gross domestic product (GDP)	Gridded GDP	2005, 2015	1 km	

* Considering the lag effect of environmental factors on health [39], the percentage of farmlands in 1995 and 2005 are used as the corresponding environmental risk factor in period I and II, respectively.

**Table 2:** Determinants of GCM spatial heterogeneity.

	Human-Made Pollution		Physical Environment	Socioeconomic Level	
proxy variables	distance from river	the percentage of farmlands	elevation	population density	GDP
q statistic (Period I)	0.15 ***	0.00	0.00	0.00	0.00
q statistic (Period II)	0.11 ***	0.03 ***	0.00	0.02 ***	0.03 ***
Interaction q statistic (Period II)	0.14 ***		0.00	0.04 ***	

*** The result is statistically significant at 0.05 level.

**Table 3:** Average GCM in each region.

Proxy Variables	Distance from River (km)			Percentage of Farmlands (%)		Elevation (m)		Population Density (per/km²)		GDP (10,000 Yuan/km²)	
Strata	<3	3–10	10–15	≤median	>median	≤median	>median	≤median	>median	≤median	>median
Mean GCM (Period I, 1/10^5^)	31.38	26.09	19.56	Not significant							
Mean GCM (Period II, 1/10^5^)	22.74	26.93	32.44	24.60	27.47	Not significant		27.17	24.72	27.46	24.51

The result is statistically significant at 0.05 level.

## Supplementary Materials

The following are available online at https://www.mdpi.com/1660-4601/16/5/784/s1. Table S1: Surface water quality in S County from 2004–2015.

## References

[1] Chen W., Zheng R., Baade P.D., Zhang S., Zeng H., Bray F., Jemal A., Yu X.Q., He J. (2016). Cancer statistics in China, 2015. CA Cancer J. Clin..

[2] Huang H.-Y., Shi J.-F., Guo L.-W., Zhu X.-Y., Wang L., Liao X.-Z., Liu G.-X., Bai Y.-N., Mao A.-Y., Ren J.-S. (2016). Expenditure and financial burden for common cancers in China: a hospital-based multicentre cross-sectional study. Lancet.

[3] Zhang S. (2008). Meta-analysis of the relationship between dietary habits and gastric cancer of Chinese residents. Mod. Prev. Med..

[4] Fang X., Wei J., He X., An P., Wang H., Jiang L., Shao D., Liang H., Li Y., Wang F. (2015). Landscape of dietary factors associated with risk of gastric cancer: A systematic review and dose-response meta-analysis of prospective cohort studies. Eur. J. Cancer.

[5] Rota M., Pelucchi C., Bertuccio P., Matsuo K., Zhang Z.F., Ito H., Hu J., Johnson K.C., Palli D., Ferraroni M. (2017). Alcohol consumption and gastric cancer risk-A pooled analysis within the StoP project consortium. Int. J. Cancer.

[6] Tsugane S. (2005). Salt, salted food intake, and risk of gastric cancer: epidemiologic evidence. Cancer Sci..

[7] Yang G.H., Zhuang D.F. (2014). Atlas of the Huai River Basin Water Environment: Digestive Cancer Mortality.

[8] Ren H.Y., Wan X., Yang F., Shi X., Xu J., Zhuang D., Yang G. (2014). Association between changing mortality of digestive tract cancers and water pollution: a case study in the Huai River Basin, China. Int. J. Environ. Res. Public Health.

[9] Zhao Q., Wang Y., Cao Y., Chen A., Ren M., Ge Y., Yu Z., Wan S., Hu A., Bo Q. (2014). Potential health risks of heavy metals in cultivated topsoil and grain, including correlations with human primary liver, lung and gastric cancer, in Anhui province, Eastern China. Sci. Total Environ..

[10] Plummer M., Franceschi S., Vignat J., Forman D., de Martel C. (2015). Global burden of gastric cancer attributable to Helicobacter pylori. Int. J. Cancer.

[11] Fock K.M., Talley N., Moayyedi P., Hunt R., Azuma T., Sugano K., Xiao S.D., Lam S.K., Goh K.L., Chiba T. (2008). Asia–Pacific consensus guidelines on gastric cancer prevention. J. Gastroenterol. Hepatol..

[12] Wang J., Chengdong X.U. (2017). Geodetector: Principle and prospective. Acta Geogr. Sin..

[13] Tobler W.R. (1970). A computer movie simulating urban growth in the Detroit region. Econ. Geogr..

[14] Wang J.F., Li X.H., Christakos G., Liao Y.L., Zhang T., Gu X., Zheng X.Y. (2010). Geographical Detectors-Based Health Risk Assessment and its Application in the Neural Tube Defects Study of the Heshun Region, China. Int. J. Geogr. Inf. Sci..

[15] Fei X., Wu J., Liu Q., Ren Y., Lou Z. (2015). Spatiotemporal analysis and risk assessment of thyroid cancer in Hangzhou, China. Stoch. Environ. Res. Risk Assess..

[16] Huang J., Wang J., Bo Y., Xu C., Hu M., Huang D. (2014). Identification of health risks of hand, foot and mouth disease in China using the geographical detector technique. Int. J. Environ. Res. Public Health.

[17] Wang J.F., Wang Y., Zhang J., Christakos G., Sun J.L., Liu X., Lu L., Fu X.Q., Shi Y.Q., Li X.M. (2013). Spatiotemporal transmission and determinants of typhoid and paratyphoid fever in Hongta District, Yunnan Province, China. PLoS Negl. Trop. Dis..

[18] Li Y., Li H., Liu Z., Miao C. (2016). Spatial Assessment of Cancer Incidences and the Risks of Industrial Wastewater Emission in China. Sustainability.

[19] Liu L. (2010). Made in China: Cancer Villages. Environ. Sci. Policy Sustain. Dev..

[20] Zhang K.M., Wen Z.G. (2008). Review and challenges of policies of environmental protection and sustainable development in China. J. Environ. Manag..

[21] Feng L., Liao W. (2016). Legislation, plans, and policies for prevention and control of air pollution in China: achievements, challenges, and improvements. J. Clean. Prod..

[22] Li M., Wang S., Han X., Liu W., Song J., Zhang H., Zhao J., Yang F., Tan X., Chen X. (2017). Cancer mortality trends in an industrial district of Shanghai, China, from 1974 to 2014, and projections to 2029. Oncotarget.

[23] Xi W., Xu Z. (2002). Legal control of water pollution in Huai river, China: A case study. Proceedings of the Conference Paper for Sixth International Conference on Environmental Compliance and Enforcement.

[24] Ren H., Xu D., Shi X., Xu J., Zhuang D., Yang G. (2016). Characterisation of gastric cancer and its relation to environmental factors: A case study in Shenqiu County, China. Int. J. Environ. Health Res..

[25] Zhong M. (2006). Evaluation of the implementation of water pollution prevention and control plans in China: The case of Huai River Basin. Background Paper, World Bank Policy Analytical and Advisory Assistance Program.

[26] Henan Province Arranges Special Funds to Solve Drinking Water Safety Problems in Heavily Polluted Areas. http://www.people.com.cn/GB/huanbao/1073/3192409.html.

[27] Zhou L., Jiangang X.U., Jiang J., Yuan Y., Sun D. (2013). Spatial diversity characteristics of comprehensive control ability for water environmental pollution in the Huaihe River Basin. Prog. Geogr..

[28] Wan X., Zhou M., Tao Z., Ding D., Yang G. (2011). Epidemiologic application of verbal autopsy to investigate the high occurrence of cancer along Huai River Basin, China. Popul. Health Metr..

[29] Ministry of Public Security (2014). Demographic Statistics of Counties and Cities in People’s Republic of China (2012).

[30] Richardson S., Thomson A., Best N., Elliott P. (2004). Interpreting Posterior Relative Risk Estimates in Disease-Mapping Studies. Environ. Health Perspect..

[31] Goovaerts P. (2005). Geostatistical analysis of disease data: estimation of cancer mortality risk from empirical frequencies using Poisson kriging. Int. J. Health Geogr..

[32] Clayton D., Kaldor J. (1987). Empirical Bayes estimates of age-standardized relative risks for use in disease mapping. Biometrics.

[33] Qi X., Ji W., Ren H., Guo Y., Zhou M., Yang G., Zhuang D. (2012). Model Analysis of Upper Digestive Tract Cancer and Environmental Pollution in Huaihe River Watershed. J. Geo-Inf. Sci..

[34] Quinton J.N., Catt J.A. (2007). Enrichment of heavy metals in sediment resulting from soil erosion on agricultural fields. Environ. Sci. Technol..

[35] Cohen D.A., Farley T.A., Mason K. (2003). Why is poverty unhealthy? Social and physical mediators. Soc. Sci. Med..

[36] Mosadeghrad A.M. (2014). Factors affecting medical service quality. Iran J. Public Health.

[37] FU J., JIANG D., HUANG Y. (2014). 1 km grid population dataset of China (2005, 2010). Acta Geogr. Sini..

[38] Huang Y., Jiang D., Fu J. (2014). 1 km grid GDP dataset of China (2005, 2010). Acta Geogr. Sin..

[39] Selinus O., Alloway B.J., Centeno J.A., Finkelman R.B., Fuge R., Lindh U., Smedley P. (2013). Essentials of Medical Geology.

[40] Anselin L., Getis A. (2010). Perspectives on spatial data analysis. Perspectives on Spatial Data Analysis.

[41] Liwei W., Changchun F., Shuncai X. (2014). Return intention of migrant workers in a traditional agricultural area and planning response: Based on a questionnaire survey in Zhoukou, Henan Province. Prog. Geogr..

[42] Wei J., Dafang Z., Hongyan R., Dong J., Yaohuan H., Xinliang X., Wei C., Xiaosan J. (2013). Spatiotemporal variation of surface water quality for decades: a case study of Huai River System, China. Water Sci. Technol..

[43] Dai X., Liu C., Li L. (2007). Discussion and countermeasures on safe drinking water in the rural areas of China. Acta Geogr. Sin..

[44] “Thirteenth Five-Year Plan” for Poverty alleviation in S County. http://www.shenqiu.gov.cn/news_xx.asp?msg=52393.

[45] Streeter J.L. (2017). Socioeconomic Factors Affecting Food Consumption and Nutrition in China: Empirical Evidence During the 1989–2009 Period. Chin. Econ..

[46] Go M. (2002). Natural history and epidemiology of Helicobacter pylori infection. Aliment. Pharmacol. Ther..

[47] Yang L., Zheng R., Wang N., Yuan Y., Liu S., Li H., Zhang S., Zeng H., Chen W. (2018). Incidence and mortality of stomach cancer in China, 2014. Chin. J. Cancer Res..

[48] Song P., Wu L., Guan W. (2015). Dietary Nitrates, Nitrites, and Nitrosamines Intake and the Risk of Gastric Cancer: A Meta-Analysis. Nutrients.

