# Supplementary material for: Spatiotemporal Variations in Gastric Cancer Mortality and Their Relations to Influencing Factors in S County, China
Source: Int J Environ Res Public Health. 2019 Mar 4;16(5):784. doi: 10.3390/ijerph16050784 (PMC6427783; doi:10.3390/ijerph16050784)
Supplement: Supplementary file 1 [file ijerph-16-00784-s001.pdf]

China has established a water quality classification system based on purpose of use and protection target, following Environmental Quality Standard GB3838-2002.

Grade I – Mainly applicable to the source of water bodies and national nature preserves.

Grade II – Mainly applicable to class A water source protection area for centralized drinking water supply, sanctuaries for rare species of fish, and spawning grounds for fish and shrimps.

Grade III – Mainly applicable to class B water source protection area for centralized drinking water supply, sanctuaries for common species of fish, and swimming zones.

Grade IV – Mainly applicable to water bodies for general industrial water supply and recreational waters in which there is not direct human contact with the water.

Grade V – Mainly applicable to water bodies for agricultural water supply and for general landscape requirements.

Grade V+ - Essentially useless

The surface water quality data for state-controlled stations were extracted from the series of China Environmental Quality Reports, which were published by the State Environmental Protection Administration from 2004 to 2015.

Table S1: Surface water quality in S County from 2004-2015

Year	2004	2005	2006	2007	2008	2009	2010	2011	2012	2013	2014	2015
**SY River**	V+	V+	V+	V+	V+	V+	V+	V+	NA	V	V	IV
**FQ river**	V+	V+	V+	V+	V+	V+	V	NA	NA	NA	NA	NA

'NA' means no data.
